# High Environmental Temperature Increases Glucose Requirement in the Developing Chicken Embryo

**DOI:** 10.1371/journal.pone.0059637

**Published:** 2013-04-01

**Authors:** Roos Molenaar, Joost J. G. C. van den Borne, Ewoud Hazejager, Niels B. Kristensen, Marcel J. W. Heetkamp, Ron Meijerhof, Bas Kemp, Henry van den Brand

**Affiliations:** 1 HatchTech B.V., Veenendaal, The Netherlands; 2 Animal Nutrition Group, Wageningen University, Wageningen, The Netherlands; 3 Adaptation Physiology Group, Wageningen University, Wageningen, The Netherlands; 4 Knowledge Centre for Agriculture, Aarhus N, Denmark; 5 Poultry Performance Plus, Voorst, The Netherlands; Bambino Gesu’ Children Hospital, Italy

## Abstract

Environmental conditions during the perinatal period influence metabolic and developmental processes in mammals and avian species, which could impact pre- and postnatal survival and development. The current study investigated the effect of eggshell temperature (EST) on glucose metabolism in broiler chicken embryos. Broiler eggs were incubated at a high (38.9°C) or normal (37.8°C) EST from day 10.5 of incubation onward and were injected with a bolus of [U-^13^C]glucose in the chorio-allantoic fluid at day 17.5 of incubation. After [U-^13^C]glucose administration,^ 13^C enrichment was determined in intermediate pools and end-products of glucose metabolism. Oxidation of labeled glucose occurred for approximately 3 days after injection. Glucose oxidation was higher in the high than in the normal EST treatment from day 17.6 until 17.8 of incubation. The overall recovery of ^13^CO_2_ tended to be 4.7% higher in the high than in the normal EST treatment. An increase in EST (38.9°C vs 37.8°C) increased ^13^C enrichment in plasma lactate at day 17.8 of incubation and ^13^C in hepatic glycogen at day 18.8 of incubation. Furthermore, high compared to normal EST resulted in a lower yolk-free body mass at day 20.9 (−2.74 g) and 21.7 (−3.81 g) of incubation, a lower hepatic glycogen concentration at day 18.2 (−4.37 mg/g) and 18.8 (−4.59 mg/g) of incubation, and a higher plasma uric acid concentration (+2.8 mg/mL/+43%) at day 21.6 of incubation. These results indicate that the glucose oxidation pattern is relatively slow, but the intensity increased consistently with an increase in developmental stage of the embryo. High environmental temperatures in the perinatal period of chicken embryos increased glucose oxidation and decreased hepatic glycogen prior to the hatching process. This may limit glucose availability for successful hatching and could impact body development, probably by increased gluconeogenesis from glucogenic amino acids to allow anaerobic glycolysis.

## Introduction

Environmental conditions influence metabolic and developmental processes during the perinatal period of mammals and avian species [1], [2], [3]. Changes in metabolism and development in this period can have long-term effects in later life [2], [4]. Particularly glucose metabolism seems to play a crucial role in survival and early development of animals [1], [5], [6]. In chicken embryos, several studies have indicated that high environmental temperatures affect glucose metabolism and possibly explain decreases in survival and development in the peri- and posthatch period [3], [5], [7], [8].

During incubation of chicken embryos, nutrient utilization and metabolic pathways depend on the developmental stage of the embryo. Carbohydrates are present in very low levels in the egg (<1%) at oviposition and the total amount decreases even further throughout incubation [9]. A major portion of the glucose is used in the first week of incubation for anaerobic glycolysis, as the chorioallantoic membrane (CAM) is not sufficiently developed to provide enough oxygen for complete fatty acid oxidation [10], [11]. Fatty acids are the predominant energy source of chicken embryos during the major part of incubation, but glucose becomes an indispensable substrate for ATP production at the end of incubation when the hatching process occurs [12], [13]. The hatching process is energy-demanding and, the oxygen supply is restricted by limited gas exchange across the eggshell and CAM at the start of the hatching process [14], [15]. Because muscle activity is high and oxygen availability is relatively low, glucose is used for anaerobic glycolysis in muscles, resulting in increased plasma lactate concentrations [11], [12], [13], [16], [17]. Plasma glucose concentrations increase just before the hatching process starts, probably to ensure normal activity of the central nervous system [18] and to emerge successfully from the eggshell. This increase in glucose is a result of the mobilization of hepatic glycogen [12], [18], [19], [20]. Because glucose is hardly available in the egg at the start of incubation (<1%) [9], glycogen stores are built up during the incubation period in the heart, liver, muscle, and yolk sac membrane [19], [20], [21], [22].

We previously showed that hepatic glycogen stores at day 18 of incubation, just before the hatching process started, were reduced by 30% when increasing the eggshell temperature (EST) from 37.8 to 38.9°C from day 7 of incubation onward [3]. These lower hepatic glycogen levels may indicate that hepatic glycogen synthesis was lower and/or that glucose oxidation was higher in embryos incubated at high compared with normal EST.

In the current study we investigated the hypothesis that glucose oxidation is increased when chicken embryos are incubated at a high compared to normal EST. The chicken embryo is a good model to study effects of environmental conditions on metabolic processes and development in the perinatal period, because it grows without direct maternal influences. In mammals, such studies are usually complicated because the fetus is connected to and influenced by the mother. In the current study, a novel approach was developed in a series of experiments to quantify glucose partitioning between anabolic and catabolic processes during late embryonic development in the avian embryo. Chicken embryos, incubated at a normal or high EST, were injected with [U-^13^C]glucose in the chorio-allantoic fluid at day 17.5 of incubation. The metabolic fate of labeled glucose was assessed by measuring ^13^C recovery in expired CO_2_, plasma glucose and lactate, and hepatic glycogen.

## Methods

### Design and Measurements Study I and II

Two studies were conducted to develop the technique to measure oxidation of [U-^13^C]glucose after injection into the chorio-allantoic fluid of chicken embryos. In addition, a contrast in EST was created in each of the two studies to provide general insight into the effects of environmental temperature on glucose oxidation in chicken embryos in the last week of incubation. For both studies, 150 fertile broiler eggs at day 13 of incubation were obtained from a Ross 308 flock of 59 weeks of age (Morren B.V., Lunteren, the Netherlands). Eggs were randomly divided between two identical open-circuit climate respiration chambers (CRC) [23] and incubated at a normal (37.8°C; n = 75) or a high (38.9°C; n = 75) EST. The normal and high EST values were based on studies [24], [25], because these studies showed that an EST of 38.9°C negatively influenced hatchability and body development. Eggshell temperature was regulated in the CRC as described by [23], [25] and relative humidity was maintained at 55%. Eggs were turned each hour over 90°. In study I, a solution containing [U-^13^C]glucose (99 atom% ^13^C, Sigma-Aldrich Chemie B.V., Zwijndrecht, the Netherlands; 1.0 mg in 250 µL of sterile water) was injected as a single bolus into the chorio-allantoic fluid in each of the 150 eggs at day 14.5 of incubation. Eggs at the normal EST treatment were injected on average 1.5 hours earlier than eggs at the high EST treatment. In study II, a solution containing [U-^13^C]glucose (Sigma-Aldrich Chemie B.V.; 0.73 mg in 250 µL of sterile water) was repeatedly injected as a bolus into the chorio-allantoic fluid in each of the 150 eggs for 4 consecutive days from day 14.5 of incubation onward.

For *in ovo* [U-^13^C]glucose injection, the air cell was located by candling the egg. The blunt end of each egg was sterilized with 70% ethanol. A 20-gauge needle was punctured through the eggshell at 2 to 3 mm above the air cell. A 25-gauge needle was inserted for ±7 mm through the hole to enter the chorioallantoic fluid and to inject the [U-^13^C]glucose solution. The injection hole was sealed with liquid paraffin. In both studies, total CO_2_ and ^13^CO_2_ production were measured every 6 minutes as described by [26] from day 13 until 18 of incubation. ^13^C enrichment in expired CO_2_ was expressed as a percentage of total CO_2_ production (atom% ^13^C). The natural background of ^13^C in expired CO_2_ was measured during 1.5 days prior to the [U-^13^C]glucose injection. Background ^13^C enrichment was subtracted from ^13^C enrichment and expressed as atom percentage excess (APE).

### Design Study III

The main study was designed to investigate effects of environmental temperature on glucose metabolism in chicken embryos during the perinatal period. A normal (37.8°C) or high (38.9°C) EST treatment was applied from day 10.5 until 21.6 of incubation in 4 subsequent batches of 160 eggs each. Fertile broiler eggs at day 10.5 of incubation were obtained from a commercial Ross 308 breeder flock aged between 42 and 44 weeks (Lagerwey B.V., Lunteren, the Netherlands). On day 10.5 of incubation, eggs weighing between 60 and 64 g were selected. Per batch, eggs were randomly distributed between the two open-circuit CRC and incubated at a normal (37.8°C; n = 80) or a high (38.9°C; n = 80) EST treatment as described for the first 2 studies.

At day 17.5 of incubation, all eggs were injected with a single bolus of [U-^13^C]glucose (1.0 mg in 250 µL of sterile water) into the chorio-allantoic fluid using the same method as described for the first two studies. After injection, eggs were transferred from an egg tray to a hatching basket (560×360 mm) in the first three batches and to individual hatching baskets (120×135 mm) in the fourth batch. The hatching baskets were placed in the same CRC and eggs were exposed to the same EST treatment as before; this EST was maintained in the CRC until day 18.5 of incubation. At day 18.5 of incubation, the environmental temperature in the CRC was fixed at the normal or high EST, and the EST was allowed to increase during the remainder of the hatching process. Eggs in the first three batches were recorded by a video camera to determine individual hatching times. The study was approved by the Institutional Animal Care and Use Committee of Wageningen University, the Netherlands.

### Measurements Study III

In the first three batches, ^13^C enrichment in expired CO_2_ was determined after injection of a single bolus of [U-^13^C]glucose (99 atom% 13C, Sigma-Aldrich Chemie B.V.; 1.0 mg in 250 µL of sterile water) into the chorio-allantoic fluid at day 17.5 of incubation. In the fourth batch, ^13^C enrichment in plasma glucose, lactate, and hepatic glycogen were determined at eight time points between day 17.5 and 21.6 of incubation, and chick quality characteristics (yolk-free body mass and yolk weight) and blood metabolites were measured. The injection of the bolus was performed at day 17.5 of incubation to include the energy-demanding hatching process which starts around day 19 of incubation, during the period that glucose has an important role as energy substrate [13], [27], [28]. Based on results of study I and II, it was concluded that glucose oxidation (i.e. ^13^C enrichment over the background) was detectable for 1 to 2.5 days after tracer administration.

In the first three batches, total CO_2_ and ^13^CO_2_ production were measured at 6 minute intervals as described by [26] from day 10.5 until 21.6 of incubation. The ^13^C enrichment in excess of the natural background of ^13^C enrichment in CO_2_ was calculated from these measurements, as described for study I and II. The background enrichment was determined by calculating the mean ^13^C enrichment in CO_2_ from day 10.5 until 17.5 of incubation. ^13^C enrichment in expired CO_2_ was expressed per hour of incubation. All chickens were euthanized with CO_2_ at 21.6 days after the start of incubation.

In the fourth batch, at eight different time points during incubation 10 animals per timepoint and EST treatment were randomly chosen to determine ^13^C enrichment in plasma glucose, plasma lactate, and hepatic glycogen. Chicken development and blood metabolites were also assessed. The time points were selected to cover the peak in ^13^CO_2_ expiration, estimated from the first three batches, and were at day 17.5, 17.8, 18.2, 18.8, 19.7, 20.4, 20.9, and 21.6 of incubation. Embryos or chickens were weighed before blood collection. Blood samples were taken from the jugular vein in embryos and after decapitation in chickens. All blood samples were collected in a 4-mL blood collection tube containing 10 mg of sodium fluoride and 8 mg of potassium oxalate (BD Vacutainer, Franklin Lakes, NJ). An extra droplet (0.02 mL) of 10% heparin was added to the collection tube prior to sampling. Blood was centrifuged (2,900×*g*) for 15 minutes and plasma was decanted and stored at −20°C until further analysis. Plasma glucose, lactate, and uric acid concentrations were determined with commercial kits (DiaSys Diagnostic Systems International, Holzheim, Germany). After bleeding, livers of embryos and chickens were immediately dissected, weighed, and frozen in liquid nitrogen. Livers were stored at −80°C until further analysis. The yolk was removed and weighed. Yolk-free body mass (YFBM) was calculated as body weight minus yolk weight. YFBM is assumed to reflect chicken development better than body weight because the latter contains an amount of residual yolk that is not metabolized yet [29].

To determine ^13^C enrichment in plasma glucose [30], 1 mL ice-cold ethanol (96%) was added to 100 µL of plasma and this mixture was stored overnight at −20°C before centrifugation (15,339×*g*) at 4°C for 20 minutes. After evaporating the supernatant to dryness, 75 µL acetic acid anhydride-pyridine (10∶5 vol/vol) was added for 1.5 hours at room temperature to convert glucose to its penta-acetate derivate. The reagent was evaporated to dryness and dissolved in 500 µL chloroform. The ^13^C/^12^C ratio was then determined by gas chromatography-combustion isotope ratio mass spectrometry (GC-C-IRMS; Delta C, Finnigan MAT, Bremen, Germany). The measured ^13^C enrichment was corrected for the dilution of C atoms during derivatization.

^13^C enrichment in plasma lactate was determined by GC-C-IRMS analysis (Delta V Plus; Finnigan MAT, Bremen, Germany) of the 2-O-ethoxycarbonyl ethyl ester of lactic acid. Atomic composition of lactic acid was obtained by correction for carbon added in derivatization using a mass balance equation as previously described [31].

To determine hepatic glycogen enrichment, the whole liver was homogenized with a glass-stirring spoon after the addition of 1 µL of 7% HCLO_4_/mg of wet tissue. The suspension was centrifuged (2,900×*g*) at 4°C for 15 minutes. The supernatant was decanted and cleaned with 1 mL of petroleum ether. To extract hepatic glycogen, 2.5 mL ice-cold ethanol (96%) was added to 400 µL supernatant for 120 minutes at −20°C. Then, the solution was centrifuged (2,900×*g*) at 0°C for 15 minutes, the fluid was removed and a glycogen pellet was left on the bottom of the tube. The glycogen pellet was dissolved in 1.025 mL water, and the glucose units were released by incubating the solution with a mixture of 25 µL amyloglucosidase (3,200 U/mL; Omnilabo, Breda, the Netherlands), 0.875 mL distilled water and 125 µL 2 M acetate buffer (2M acetic acid:2M acetate, 4∶6 vol/vol; pH = 4.8) at 60°C for 1 hour. The reaction was finished by placing the tubes on crushed ice, and 100 µL of the mixture was used to determine the ^13^C/^12^C ratio in the released glucose units by GC-C-IRMS using the procedure described for plasma glucose enrichment [30]. The measured ^13^C enrichment was corrected for the dilution of C atoms during the derivatization.

The remaining mixture was used to determine the hepatic glycogen concentration. Proteins were precipitated by adding 200 µL of Carrez I (106 g potassium hexacyanoferrate (II) trihydrate dissolved in 1 L water) plus 200 µL of Carrez II (219.5 g zinc acetate dihydrate and 30 g acetic acid dissolved in 1 L water) solution to the mixture. After centrifugation (15,339×*g*) at 4°C for 10 minutes, the supernatant was used to determine glucose concentration with a commercial kit (DiaSys Diagnostic Systems International, Holzheim, Germany). The glucose concentrations were corrected with 0.9 to obtain hepatic glycogen concentrations, expressed as mg/g wet tissue.

Enrichment in plasma glucose and lactate was corrected for the natural background that was determined in embryos samples at day 17.5 of incubation i.e. these embryos did not receive [U-^13^C] glucose, and was expressed in APE. ^13^C in hepatic glycogen was calculated in mmol over the baseline by multiplying the hepatic glycogen concentration by liver weight and by the ^13^C enrichment (in APE).

### Calculation Recovery Study III

Recovery of ^13^C in expired CO_2_ was calculated by expressing ^13^CO_2_ production in excess of the background relative to the dose injected at day 17.5 of incubation. Embryos were not included to calculate recovery of the ^13^C tracer as ^13^CO_2_ when they died during incubation (i.e. from the moment of death onwards).

### Statistical Analysis

Data from study I and II were not statistically analyzed because these studies were designed to develop the technique to measure [U-^13^C]glucose oxidation and to provide an initial insight in the effect of EST. Repeated measurements on the same treatment combination were therefore not performed. In the first three batches of study III, ^13^C enrichment in expired CO_2_ was calculated per hour and analyzed using the MIXED procedure for repeated measurements in SAS [32] (Version 9.1) with EST, day of incubation, and their interaction as fixed factors. Day of incubation was the repeated factor and an auto-regressive covariance structure was used. All other variables were analyzed using a GLM procedure with EST, day of incubation, and their interaction as fixed factors. Recovery of ^13^C in expired air was analyzed using a GLM procedure with EST treatment as a fixed effect and batch 1 to 3 as a block. CRC was the experimental unit in batches 1 to 3 and embryo or chicken was the experimental unit in batch 4. Distributions of the means and residuals were examined to check model assumptions. Least squares means were compared using Bonferroni adjustments for multiple comparisons. Data are presented as least squares means ± SEM. In all cases, a significant difference was considered at *P*≤0.05 and a tendency was considered at 0.05<*P*<0.10.

## Results

### Study I and II: ^13^C Enrichment in Expired CO_2_

**Figure 1:**
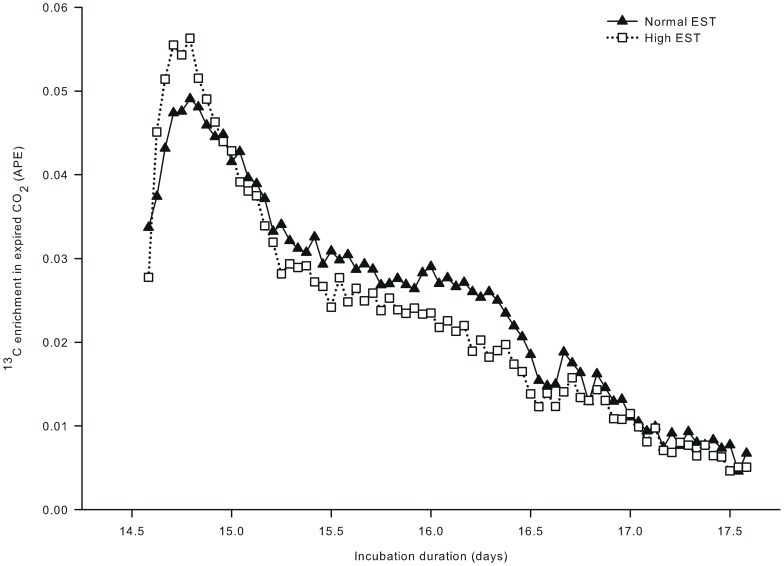
^13^C enrichment in expired CO_2_ in eggs incubated at two different eggshell temperatures (study I). ^13^C enrichment in expired CO_2_ after injecting a single bolus of [U-^13^C]glucose (1.0 mg in 250 µL sterile water) in the chorio-allantoic fluid at day 14.5 of incubation of embryos incubated at a normal (37.8°C) or high (38.9°C) eggshell temperature (EST) from day 13 of incubation onward.

In study I, a single bolus of [U-^13^C]glucose was injected at day 14.5 of incubation and ^13^C enrichment in expired ^13^CO_2_ showed a peak for both EST treatments within 8 hours after the injection (Figure 1A). The increase in ^13^C enrichment after the single bolus of [U-^13^C]glucose was numerically 13% higher at the high EST treatment compared to the normal EST treatment. From day 14.8 of incubation onward, ^13^C enrichment in expired CO_2_ decreased gradually in both EST treatments but did not return to the baseline until day 17.6 of incubation.

**Figure 2:**
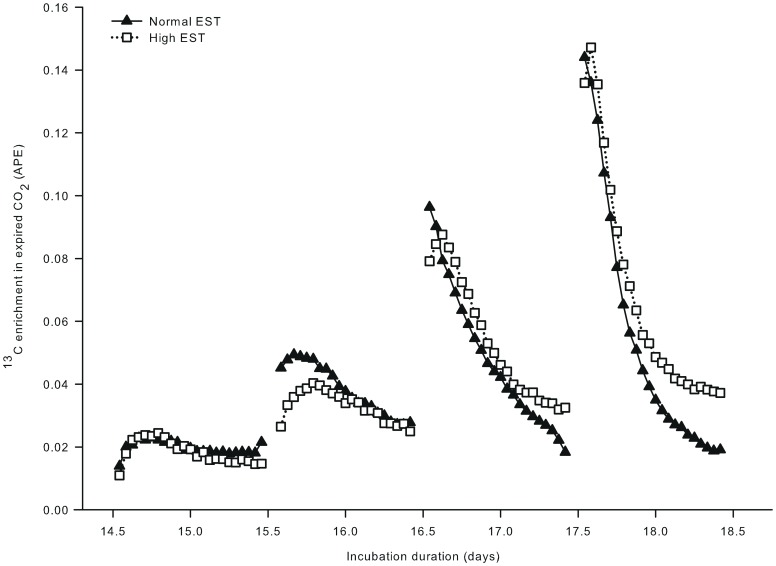
^13^C enrichment in expired CO_2_ of embryos incubated at two different eggshell temperatures (Study II). ^13^C enrichment in expired CO_2_, after injecting a single bolus of [U-^13^C]glucose (0.73 mg in 250 µL sterile water) for four consecutive days from day 14.5 of incubation in the chorio-allantoic fluid of embryos incubated at a normal (37.8°C) or high (38.9°C) eggshell temperature (EST) from day 13 of incubation onward.

In study II, a bolus of [U-^13^C]glucose was injected on 4 consecutive days from day 14.5 of incubation onward. ^13^C enrichment in expired CO_2_ responded to each bolus (Figure 2), but the height of the ^13^C enrichment peak increased with each of the four injections (respectively 0.0237, 0.0449, 0.0920 and 0.1465 APE). The time to reach this maximum ^13^C enrichment decreased with each of the four injections (from approximately 5.5 to 1.5 hours). At approximately 1 day after the 1^st^ and 2^nd^ injection, the increase in ^13^C enrichment in expired CO_2_ was numerically lower at the high than at the normal EST treatment. In contrast, after the 3^rd^ and 4^th^ injection, the ^13^C enrichment in expired CO_2_ was numerically higher at the high than at the normal EST treatment.

### Study III: ^13^C Enrichment in Expired CO_2_

**Figure 3:**
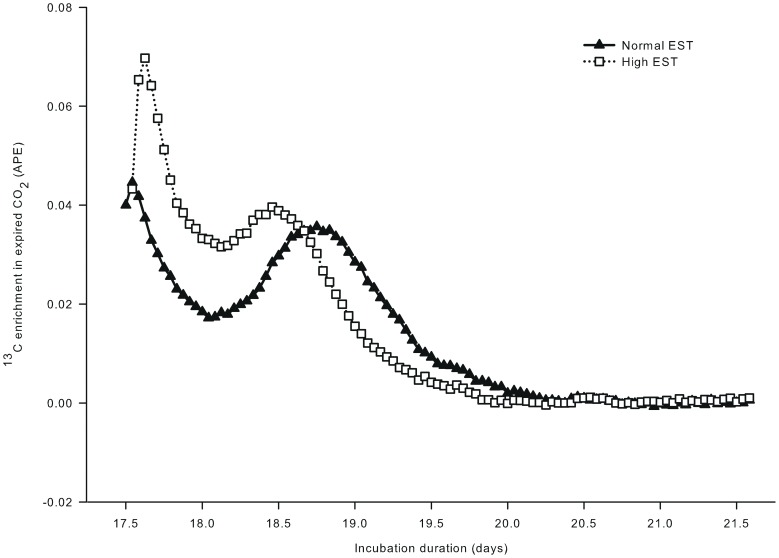
^13^C enrichment in expired CO_2_ of embryos incubated at two different eggshell temperatures (study III). ^13^C enrichment in expired CO_2_ after injecting a single bolus of [U-^13^C]glucose (1.0 mg in 250 µL sterile water) at day 17.5 of incubation in the chorio-allantoic fluid of embryos incubated at a normal (37.8°C) or high (38.9°C) eggshell temperature (EST) from day 10.5 of incubation onward. From day 17.6 until 17.8 of incubation,^ 13^C enrichment in expired CO_2_ was higher for the high EST compared to the normal EST treatment (*P*<0.05).

For both EST treatments, the ^13^C enrichment in expired CO_2_ showed a biphasic pattern after a single injection of [U-^13^C]glucose at day 17.5 of incubation (Figure 3). The first peak occurred within 3 hours after injection and this peak was approximately 36% higher in the high EST treatment than in the normal EST treatment (Δ = 0.02512; *P*<0.05). Eggs in the normal EST treatment were injected approximately 1.5 hours prior to eggs in the high EST treatment, resulting in a small time lag between the two treatments for the first peak in CO_2_ enrichment (at day 17.5 vs 17.6 of incubation, respectively). Nonetheless, the 2^nd^ peak occurred 1.3 days after the injection at the normal EST treatment and already at 0.9 days after the injection at the high EST treatment. The height of the peak did not differ between EST treatments (*P*>0.05). ^13^C enrichment in expired CO_2_ decreased with time after the 2^nd^ peak and returned to baseline at day 19.5 of incubation for the high EST treatment and at day 19.8 of incubation for the normal EST treatment (*P*>0.05).

The ^13^C enrichment in expired CO_2_ was higher at the high EST treatment than at the normal EST treatment from day 17.6 until 17.8 of incubation (*P*<0.05). Recovery of ^13^C in expired CO_2_ relative to the dose of ^13^C that was injected as glucose in the eggs tended to be higher (*P* = 0.07) at the high (46.7±0.96%) than at the normal (42.0±0.96%) EST treatment.

### Study III: ^13^C Enrichment in Plasma Glucose and Lactate, and Hepatic Glycogen

**Figure 4:**
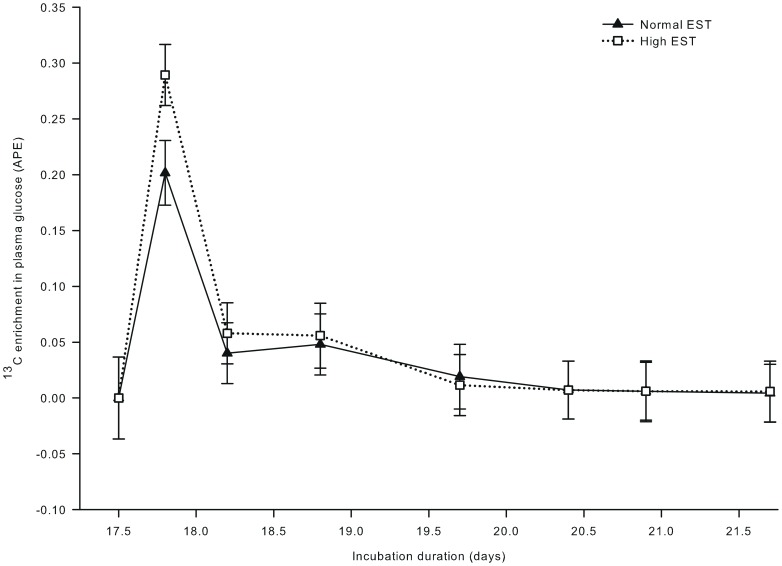
^13^C enrichment in plasma glucose of embryos incubated at two different eggshell temperatures (study III). ^13^C enrichment in plasma glucose after injecting a single bolus of [U-^13^C]glucose (1.0 mg in 250 µL sterile water) at day 17.5 of incubation in the chorio-allantoic fluid of embryos incubated at a normal (37.8°C) or high (38.9°C) eggshell temperature (EST) from day 10.5 of incubation onward. At day 17.5, 17.8, 18.2, and 18.9 of incubation, no chickens were hatched yet. At day 19.7 of incubation, 22% of the chickens of the high EST treatment and 10% of the chickens of the normal EST treatment were hatched. At day 20.4 of incubation, 80% of the chickens of the high EST treatment were hatched and all chickens of the normal EST treatment were hatched. At day 20.9 and 21.6 of incubation, all chickens had been hatched.

**Figure 5:**
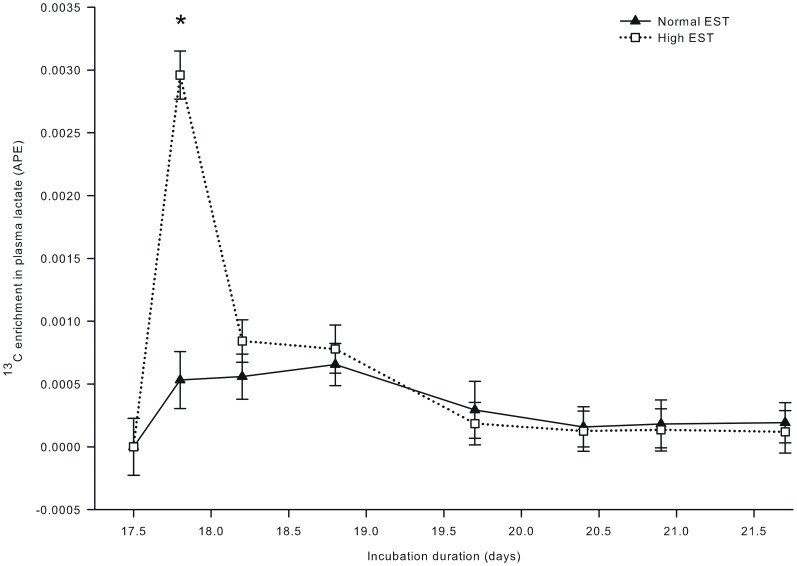
^13^C enrichment in plasma lactate of embryos incubated at two different eggshell temperatures (study III). ^13^C enrichment in plasma lactate after injecting a single bolus of [U-^13^C]glucose (1.0 mg in 250 µL sterile water) at day 17.5 of incubation in the chorio-allantoic fluid of embryos incubated at a normal (37.8°C) or high (38.9°C) eggshell temperature (EST) from day 10.5 of incubation onward. *Significant difference between EST treatments (*P*<0.05). At day 17.5, 17.8, 18.2, and 18.9 of incubation, no chickens were hatched yet. At day 19.7 of incubation, 22% of the chickens of the high EST treatment and 10% of the chickens of the normal EST treatment were hatched. At day 20.4 of incubation, 80% of the chickens of the high EST treatment were hatched and all chickens of the normal EST treatment were hatched. At day 20.9 and 21.6 of incubation, all chickens had been hatched.

**Figure 6:**
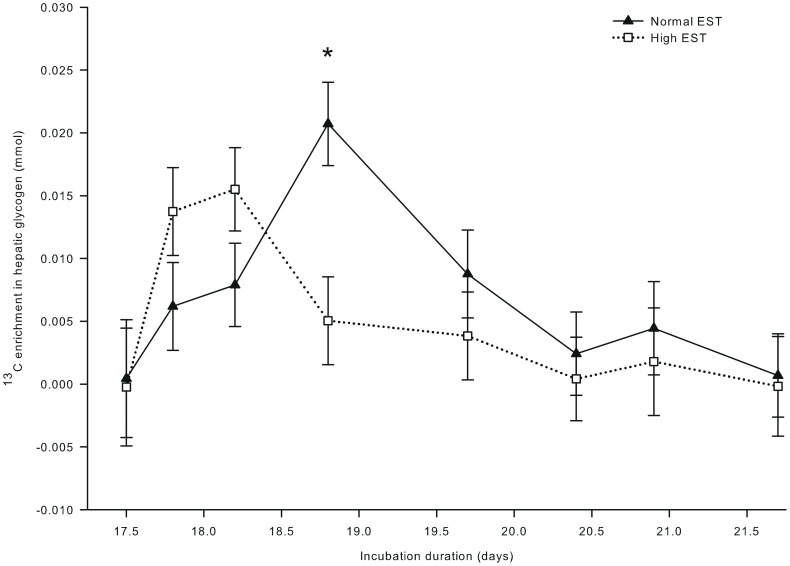
^13^C in hepatic glycogen of embryos incubated at two different eggshell temperature (study III). ^13^C in hepatic glycogen after injecting a single bolus of [U-^13^C]glucose (1.0 mg in 250 µL sterile water) at day 17.5 of incubation in the chorio-allantoic fluid of embryos incubated at a normal (37.8°C) or high (38.9°C) eggshell temperature (EST) from day 10.5 of incubation onward. *Significant difference between EST treatments (*P*<0.05). At day 17.5, 17.8, 18.2, and 18.9 of incubation, no chickens were hatched yet. At day 19.7 of incubation, 22% of the chickens of the high EST treatment and 10% of the chickens of the normal EST treatment were hatched. At day 20.4 of incubation, 80% of the chickens of the high EST treatment were hatched and all chickens of the normal EST treatment were hatched. At day 20.9 and 21.6 of incubation, all chickens had been hatched.

^13^C enrichment in plasma glucose (Figure 4) showed a single peak at day 17.8 of incubation, and was not affected by EST (*P* = 0.35). ^13^C enrichment in plasma lactate was 82% higher at the high than at the normal EST treatment at day 17.8 of incubation (interaction EST × day of incubation; *P*<0.001; Figure 5). For both EST treatments, ^13^C enrichment in plasma lactate exceeded the background enrichment until day 18.8 of incubation. ^13^C in hepatic glycogen was lower at the high EST than at the normal EST treatment at day 18.8 of incubation (interaction EST×day of incubation; *P*<0.05; Figure 6). ^13^C in hepatic glycogen was higher at day 18.8 than at day 17.5 of incubation in the normal EST treatment.

### Study III: Blood Metabolites and Liver Glycogen

**Figure 7:**
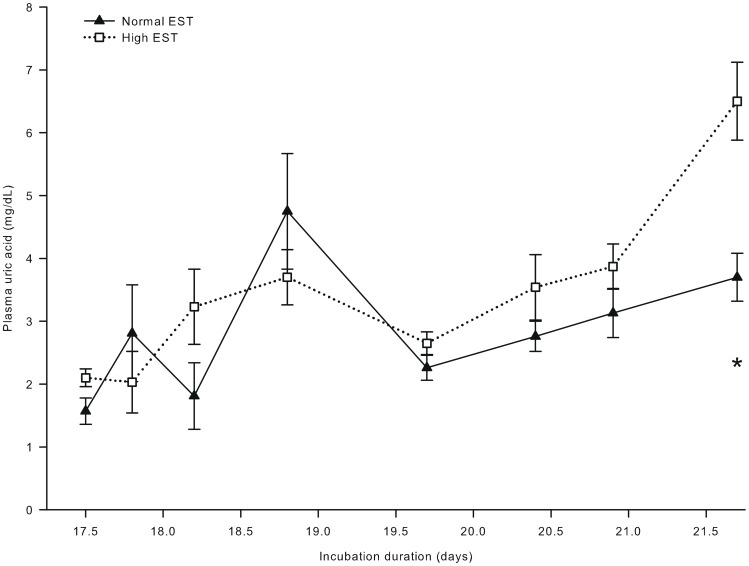
Plasma uric acid concentration of embryos incubated at two different eggshell temperatures (study III). Plasma uric acid concentration of embryos incubated at a normal (37.8°C) or high (38.9°C) eggshell temperature (EST) from day 10.5 of incubation onward. *Significant difference between EST treatments (*P*<0.05). At day 17.5, 17.8, 18.2, and 18.9 of incubation, no chickens were hatched yet. At day 19.7 of incubation, 22% of the chickens of the high EST treatment and 10% of the chickens of the normal EST treatment were hatched. At day 20.4 of incubation, 80% of the chickens of the high EST treatment were hatched and all chickens of the normal EST treatment were hatched. At day 20.9 and 21.6 of incubation, all chickens had been hatched.

**Figure 8:**
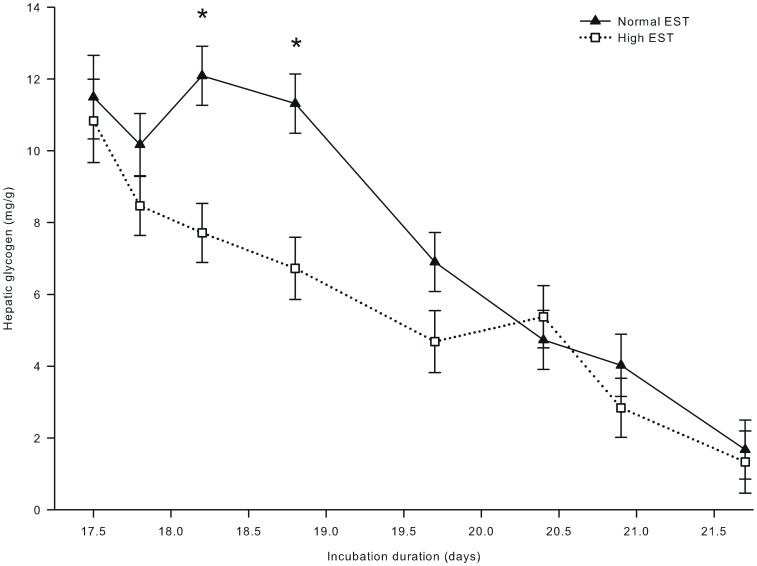
Hepatic glycogen concentration of embryos incubated at two different eggshell temperatures (study III). Hepatic glycogen concentration of embryos incubated at a normal (37.8°C) or high (38.9°C) eggshell temperature (EST) from day 10.5 of incubation onward. *Significant difference between EST treatments (*P*<0.05). At day 17.5, 17.8, 18.2, and 18.9 of incubation, no chickens were hatched yet. At day 19.7 of incubation, 22% of the chickens of the high EST treatment and 10% of the chickens of the normal EST treatment were hatched. At day 20.4 of incubation, 80% of the chickens of the high EST treatment were hatched and all chickens of the normal EST treatment were hatched. At day 20.9 and 21.6 of incubation, all chickens had been hatched.

Plasma glucose and lactate concentrations were not affected by EST (both *P*>0.50), but both increased with time of incubation (*P*<0.001). Plasma glucose concentrations increased from 172.1 mg/dL at day 17.5 to 223.1 mg/dL at day 21.6 of incubation. Plasma lactate concentrations increased from 18.4 mg/dL at day 17.5 to 45.9 mg/dL at day 20.9, and decreased thereafter to 35.7 mg/dL at day 21.6 of incubation (*P*<0.05; data not shown). Plasma uric acid concentrations were 43% higher for chickens incubated at a high than at a normal EST treatment at day 21.6 of incubation (Δ = 2.8 mg/mL; interaction EST × day of incubation; *P*<0.05; Figure 7). Hepatic glycogen concentrations were lower for chickens incubated at a high than at a normal EST treatment at day 18.2 of incubation (Δ = 4.37 mg/g) and at day 18.8 of incubation (Δ = 4.59 mg/g; interaction EST × day of incubation; *P*<0.05; Figure 8).

### Study III: Embryo and Chicken Characteristics

At day 17.5, 17.8, 18.2, and 18.9 of incubation, no chickens were hatched yet. At day 19.7 of incubation, 22% of the chickens of the high EST treatment and 10% of the chickens of the normal EST treatment had hatched. At day 20.4 of incubation, 80% of the chickens of the high EST treatment were hatched and all chickens of the normal EST treatment were hatched. At day 20.9 and 21.6 of incubation, all chickens had been hatched.

**Figure 9:**
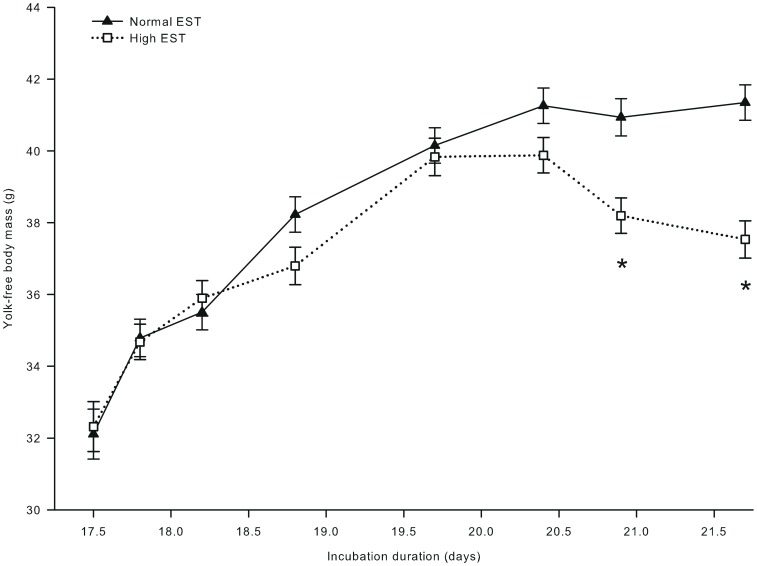
Embryo and chicken yolk-free body mass during incubation at two different eggshell temperatures (study III). Yolk-free body mass of embryos incubated at a normal (37.8°C) or high (38.9°C) eggshell temperature (EST) from day 10.5 of incubation onward. *Significant difference between EST treatments (*P*<0.05). At day 17.5, 17.8, 18.2, and 18.9 of incubation, no chickens were hatched yet. At day 19.7 of incubation, 22% of the chickens of the high EST treatment and 10% of the chickens of the normal EST treatment were hatched. At day 20.4 of incubation, 80% of the chickens of the high EST treatment were hatched and all chickens of the normal EST treatment were hatched. At day 20.9 and 21.6 of incubation, all chickens had been hatched.

Eggshell temperature did not affect YFBM until day 20.4 of incubation. YFBM was lower at the high than at the normal EST treatment at day 20.9 (Δ = 2.74 g) and day 21.6 (Δ = 3.81 g) of incubation (interaction EST × day of incubation; *P*<0.001; Figure 9). Yolk weight decreased from 12.5 g to 4.2 g from day 17.5 to 21.6 of incubation (*P*<0.001), but this decrease was not affected by EST (*P*>0.10). Liver weight increased during incubation and was 0.112 g lower at the high EST than at the normal EST treatment at day 21.6 of incubation (interaction EST × day of incubation; *P*<0.05; data not shown).

## Discussion

### Pattern of Glucose Oxidation in Chicken Embryos

To our knowledge, this is the first study that describes the pattern of glucose oxidation in the chicken embryo, which is a unique model to study embryonic metabolism without maternal influences. Glucose oxidation in the chicken embryo was detected between 48 and 72 hours after injection of a single bolus of [U-^13^C]glucose in the chorio-allantoic fluid. This is three times longer than commonly found in other species in their postnatal life. [U-^13^C]glucose is normally completely oxidized within 20 hours after an oral bolus in human infants [33] and adults [34], mice [35], rats [36], milk-fed calves [37], pigs [38], and goats [39]. In male chickens, oxidation of dietary [U-^13^C]glucose was completed approximately 10 hours after oral administration [40]. The relatively low rate of glucose oxidation in chicken embryos may be explained by the stage of development. Chicken embryos may have a lower glucose entry rate into the systemic circulation because the glucose supply predominantly originates from gluconeogenesis [11], [13], [41], whereas the majority of the glucose supply in growing and adult animals originates from exogenous (i.e. dietary) sources. Whole body glucose turnover might be reduced in chicken embryos compared to animals in their postnatal life. The longer period of glucose oxidation in chicken embryos can also be related with the absorption rate or other characteristics of the extra-embryonic allantoic fluid.

Interestingly, glucose oxidation in the current study was characterized by a biphasic pattern when the bolus was injected at day 17.5 of incubation (study III) but not when the injection was applied at day 14.5 of incubation (study I). The second peak in glucose oxidation may be related with the uptake of glucose from the allantoic fluid prior to the hatching process. The allantoic fluid is gradually absorbed from day 13 until 20 of incubation [42]. A study in the Australorp chicken embryo showed that a large portion of the allantoic fluid was absorbed between day 18 and 19 of incubation with a concurrent increase in blood glucose concentration [43]. The increased glucose uptake from the allantoic fluid occurs simultaneously with the higher glucose requirement around day 19 of incubation when the hatching process starts [11], [13]. The hatching process is energy-demanding and coincides with marginal oxygen availability because of the limited gas exchange of the eggshell conductance and the chorio-allantoic membrane [11], [14], [44]. Therefore, active muscles require anaerobic glycolysis to meet the high physical requirements [11], [12], [18] and glucose is an important ATP-yielding substrate during the hatching process [13], [27], [28]. Glucose is mainly generated from glycogen stores [11], [12], [18] as shown by the decrease in hepatic glycogen in the current study as well, but glucose absorbed from the allantoic fluid may also contribute during the hatching process. Combining the [U-^13^C]glucose tracer with an appropriate tracer for glucose uptake from the allantoic fluid would allow further refinement of the tracer technique and provide better insight in the biphasic oxidation pattern in the avian embryo.

In addition, the chicken embryo may deaminate glucogenic amino acids during the hatching process which are either immediately oxidized for ATP production [45] or first converted to glucose by gluconeogenesis [46] to allow anaerobic glycolysis. The higher plasma uric acid concentrations in the normal EST treatment at day 18.8 compared to 17.5 of incubation may suggest that gluconeogenesis of glucogenic amino acids may increase around the start of the hatching process. This was less pronounced in the high EST treatment, but this may be related with the sampling time and the physiological status of the embryo or chicken.

### Glucose Oxidation during Embryonic Development

In study II, the rate of glucose oxidation increased with each consecutive injection and the time to reach the maximum rate of oxidation decreased. Technically, this could be related to preferential oxidation of ^13^C labeled glucose, because repeated administration of the tracer may increase substrate-induced oxidation. However, the total amount of exogenous glucose was only 0.73 mg in each of the 4 boluses, which accounts for 0.18% of the average glucose pool (∼415 mg) in a 60 g egg between day 14 and 17 of incubation [43]. When such small amounts of glucose are administered, it is unlikely that the tracer administration has influenced embryonic glucose metabolism (in accordance with [47]). This suggests that the consistent increase in glucose oxidation reflects an increase in glucose requirements during embryonic development. Our findings correspond with the increase in glycolysis in muscle and liver during embryonic development [27]. Furthermore, gluconeogenesis and glycogenesis commonly increase during the second half of incubation [48], [49], [50], [51], indicating that total glucose metabolism is upregulated towards hatching.

### Glucose Oxidation at High Embryonic Temperatures

Our hypothesis was that the lower hepatic glycogen stores found in previous studies after high EST incubation [3], [52] could be explained by higher glucose oxidation. Indeed, glucose oxidation indicated by the recovery of ^13^C from glucose tended to be 4.7% higher at the high than at the normal EST treatment, whereas ^13^C enrichment in hepatic glycogen was lower at the high than at the normal EST treatment at day 18.8 of incubation. These results suggest that embryos incubated at a high EST oxidize more glucose than embryos incubated at a normal EST.

Increased glucose oxidation at the high EST treatment probably explains the lower hepatic glycogen stores at day 18.2 and 18.8 of incubation in the high EST embryos. These findings correspond with previous studies in which embryos incubated at high temperatures had reduced amounts of hepatic glycogen just before the hatching process started [3], [52]. When the amount of hepatic glycogen becomes too low, embryos may not survive the hatching process [7], [8], [24], [53], [54].

### Glucose Oxidation and Body Development

The higher plasma uric acid concentrations in the high EST chickens at day 21.6 of incubation. indicate that glucogenic amino acids were deaminated. Our results are consistent with a previous study that showed that a high EST (38.9°C vs 37.8°C) was associated with higher plasma uric acid concentrations and a lower efficiency in protein utilization for growth in chicken embryos [55]. The effects of a high EST on glucose and protein metabolism may explain the decrease in YFBM that was found at day 20.8 and 21.6 of incubation in the current study and in many other studies [7], [8], [24], [25], [56], [57].

### Anaerobic Glucose Metabolism

We hypothesized that anaerobic glycolysis is higher at a high than at a normal EST treatment because of a higher metabolic rate. Although plasma lactate concentrations were similar for both EST treatments, ^13^C enrichment in plasma lactate was substantially higher (+82%) for the high compared to the normal EST treatment directly after the [U-^13^C]glucose injection, i.e. at day 17.8 of incubation. This indicates an increased flux of ^13^C labeled glucose through anaerobic glycolysis for embryos incubated at a high compared to normal EST, and confirms our hypothesis. The absence of a difference in plasma lactate concentrations may be explained by an increased clearance rate of lactate that compensates for a higher production rate (for detailed discussion: [58]). Similarly, a study of [59] reported that lactate recycling increased at high temperatures during the early hatching phase in turkey embryos.

In conclusion, the current study showed that glucose oxidation is relatively slow in chicken embryos but increases consistently with the developmental stage of the embryo. Glucose oxidation in chicken embryos shows a biphasic pattern in the perinatal period. A high environmental temperature in the perinatal period increases the glucose requirement in chicken embryos, as indicated by an increase in glucose oxidation and a decrease in hepatic glycogen, Because of the limited glucose availability in the avian egg, the increased requirement may jeopardize successful hatching and impact body development, possibly mediated by increased gluconeogenesis from glucogenic amino acids to allow anaerobic glycolysis.
